# Supplementary material for: A giant virus forms a specialized subcellular environment within its amoeba host for efficient translation
Source: Nat Microbiol. 2026 Jan 9;11(2):584–96. doi: 10.1038/s41564-025-02234-x (PMC12872441; doi:10.1038/s41564-025-02234-x)
Supplement: Supplementary file 1 — Supplementary Tables 1–4. [file 41564_2025_2234_MOESM1_ESM.pdf]

nature microbiology

Supplementary information https://doi.org/10.1038/s41564-025-02234-x

A giant virus forms a specialized subcellular environment within its amoeba host for efficient translation

In the format provided by the authors and unedited

Supplementary Information

Supplementary Table 1. Number of reads yielded in each sample.

Time point	Replicate 1 (GB)	Replicate 2 (GB)	Library
0 hpi	1.27	1.65	Ribosome profiling
2 hpi	1.42	1.20	
4 hpi	1.57	0.98	
8 hpi	1.26	1.74	
0 hpi	8.16	7.22	RNA-Seq
2 hpi	8.88	7.11	
4 hpi	8.66	8.68	
8 hpi	6.69	7.24	
0 hpi	2.11	1.99	mim-tRNA-Seq
2 hpi	2.61	2.19	
4 hpi	1.98	1.93	
8 hpi	2.99	2.68	

Supplementary Table 2. KEGG pathway enrichment analysis done by clusterProfiler. This table was used for extended data figure 2R. Due to table size limitation, please check source data for extended data figure 2R for detailed genes in each pathway.

Cluster	ID	Description	GeneRatio	BgRatio	pvalue	p.adjust	qvalue	Count
c1	ko03010	Ribosome	36/200	93/1111	6.29e-07	3.65e-05	3.18e-05	36
c1	ko00190	Oxidative	15/200	27/1111	9.54e-06	0.000277	0.000241	15
c1	ko00020	Citrate	15/200	36/1111	0.000649	0.0125	0.0109	15
c1	ko00270	Cysteine	12/200	28/1111	0.00172	0.0250	0.0218	12
c1	ko00330	Arginine	8/200	16/1111	0.00332	0.0385	0.0335	8
c1	ko04814	Motor	14/200	38/1111	0.00400	0.0386	0.0336	14
c2	ko00970	Aminoacyl-tRNA	11/110	39/1111	0.000842	0.0238	0.0223	11
c2	ko04141	Protein	11/110	41/1111	0.00134	0.0238	0.0223	11
c2	ko03050	Proteasome	9/110	30/1111	0.00159	0.0238	0.0223	9
c3	ko03050	Proteasome	17/113	30/1111	1.65e-10	7.08e-09	6.93e-09	17
c3	ko03010	Ribosome	21/113	93/1111	0.000179	0.00385	0.00377	21
c4	ko03010	Ribosome	12/40	93/1111	4.61e-05	0.00134	0.00126	12

Supplementary Table 3. A-site offset for ribosome footprints of amoeba and APMV mRNAs.

Amoeba		APMV	
**Length**	**A-site offset**	**Length**	**A-site offset**
		18	12
19	15	19	13
20	15	20	14
21	15	21	15
22	16	22	16
23	16	23	9
25	11	24	10
26	12	25	11
27	15	26	12
28	15	27	13
29	15	28	14
30	15	29	15
31	16	30	15
32	16	31	16
		32	16

Supplementary Table 4. Exact *p*-value in all figures

Figure	Time point	*p*-value		Method
		R1	R2	
Figure 1D	2 hpi	< 2.2e-16	< 2.2e-16	Wilcoxon rank sum test (two-sided)
Figure 1D	4 hpi	< 2.2e-16	< 2.2e-16	
Figure 1D	8 hpi	< 2.2e-16	< 2.2e-16	
Figure 1E	2 hpi	< 2.2e-16	< 2.2e-16	Wilcoxon rank sum test (two-sided)
Figure 1E	4 hpi	< 2.2e-16	< 2.2e-16	
Figure 1E	8 hpi	< 2.2e-16	< 2.2e-16	
Figure 1H	2 hpi	0.56		Wilcoxon rank sum test (one-sided)
Figure 1H	4 hpi	2.4e-06		
Figure 1H	8 hpi	< 2.2e-16		
Figure 2D	2 hpi	9.6e-05	1.6e-08	Wilcoxon rank sum test (one-sided)
Figure 2D	4 hpi	0.00031	0.00044	
Figure 2D	8 hpi	0.069	0.0017	
Figure 3B	2 hpi vs 0 hpi	7.8e-44	1.1e-44	Spearman's correlation (two-tailed)
Figure 3B	4 hpi vs 0 hpi	7.6e-46	2.0e-36	
Figure 3B	8 hpi vs 0 hpi	2.5e-25	2.1e-29	
Figure 3C	0 hpi	0.0587	0.0450	Wilcoxon rank sum test (one-sided)
Figure 3C	2 hpi	0.0587	0.0457	
Figure 3C	4 hpi	0.0587	0.0464	
Figure 3C	8 hpi	0.0696	0.0580	
Figure 3D	0 hpi	2.4e-06	2.4e-06	Wilcoxon rank sum test (one-sided)
Figure 3D	2 hpi	0.51	0.36	
Figure 3D	4 hpi	0.018	0.0074	
Figure 3D	8 hpi	0.0011	3.1e-06	
Figure 4A	0 hpi	0.00021	0.0003	Wilcoxon rank sum test (one-sided)
Figure 4A	2 hpi	0.0013	0.0019	
Figure 4A	4 hpi	0.0047	0.0013	
Figure 4A	8 hpi	0.042	0.0019	
Extended 1B	Not applicable	3.4e-07		Spearman's correlation (two-tailed)
Extended 1C	Not applicable	0.03204		Spearman's correlation (two-tailed)
Extended 1D	Not applicable	0.3064		Spearman's correlation (two-tailed)
Extended 3A	2 hpi (mean+ 1 s.d.)	0.0087	0.028	Wilcoxon rank sum test (one-sided)
Extended 3A	4 hpi (mean+ 1 s.d.)	9e-05	0.0068	
Extended 3A	8 hpi (mean+ 1 s.d.)	0.67	0.0035	
Extended 3A	2 hpi (mean+ 2 s.d.)	7.7e-06	2.6e-09	
Extended 3A	4 hpi (mean+ 2 s.d.)	6.7e-05	0.00032	
Extended 3A	8 hpi (mean+ 2 s.d.)	0.063	0.00068	
Extended 3A	2 hpi (mean+ 3 s.d.)	0.00038	0.00013	
Extended 3A	4 hpi (mean+ 3 s.d.)	5.4e-05	0.0011	
Extended 3A	8 hpi (mean+ 3 s.d.)	0.019	0.0042	
Extended 4C	0 hpi	0.00152	0.00024	Spearman's correlation (two-tailed)
Extended 5A	2 hpi	< 2.22e-16	< 2.22e-16	Spearman's correlation (two-tailed)
Extended 5A	4 hpi	< 2.22e-16	< 2.22e-16	
Extended 5A	8 hpi	9.63e-08	4.56e-06	
Extended 6A	0 hpi	8.5e-05	0.0005	Wilcoxon rank sum test (one-sided)
Extended 6A	2 hpi	0.0031	0.0073	
Extended 6A	4 hpi	0.016	0.0024	

Extended 6A	8 hpi	0.22	0.0023	
Extended 6B	2 hpi	0.5	0.54	Wilcoxon rank sum test (one-sided)
Extended 6B	4 hpi	0.36	0.3	
Extended 6B	8 hpi	0.64	0.43	
Extended 7B	Not applicable	0.057 (AT-construct)	0.014 (GC-construct)	Wilcoxon rank sum test (one-sided)
