# Supplementary material for: A giant virus forms a specialized subcellular environment within its amoeba host for efficient translation
Source: Nat Microbiol. 2026 Jan 9;11(2):584–96. doi: 10.1038/s41564-025-02234-x (PMC12872441; doi:10.1038/s41564-025-02234-x)
Supplement: Supplementary file 2 — Reporting Summary [file 41564_2025_2234_MOESM2_ESM.pdf]

nature portfolio

Corresponding author(s): Hiroyuki Ogata

Last updated by author(s): Nov 17, 2025

Reporting Summary

Nature Portfolio wishes to improve the reproducibility of the work that we publish. This form provides structure for consistency and transparency in reporting. For further information on Nature Portfolio policies, see our Editorial Policies and the Editorial Policy Checklist.

Statistics

For all statistical analyses, confirm that the following items are present in the figure legend, table legend, main text, or Methods section.

n/a	Confirmed	
☐	☒	The exact sample size (*n*) for each experimental group/condition, given as a discrete number and unit of measurement
☐	☒	A statement on whether measurements were taken from distinct samples or whether the same sample was measured repeatedly
☐	☒	The statistical test(s) used AND whether they are one- or two-sided<br>*Only common tests should be described solely by name; describe more complex techniques in the Methods section.*
☒	☐	A description of all covariates tested
☐	☒	A description of any assumptions or corrections, such as tests of normality and adjustment for multiple comparisons
☐	☒	A full description of the statistical parameters including central tendency (e.g. means) or other basic estimates (e.g. regression coefficient) AND variation (e.g. standard deviation) or associated estimates of uncertainty (e.g. confidence intervals)
☐	☒	For null hypothesis testing, the test statistic (e.g. *F*, *t*, *r*) with confidence intervals, effect sizes, degrees of freedom and *P* value noted<br>*Give P values as exact values whenever suitable.*
☒	☐	For Bayesian analysis, information on the choice of priors and Markov chain Monte Carlo settings
☒	☐	For hierarchical and complex designs, identification of the appropriate level for tests and full reporting of outcomes
☒	☐	Estimates of effect sizes (e.g. Cohen's *d*, Pearson's *r*), indicating how they were calculated

Our web collection on statistics for biologists contains articles on many of the points above.

Software and code

Policy information about availability of computer code

Data collection	Ribosome profiling, RNA-Seq, and tRNA-Seq data were collected using HiSeq X (Illumina)
Data analysis	fastp v.0.21.0, STAR v.2.7.0a, SAMtools v.1.10, fastqc v.0.12.1, fastx-trimmer v.0.0.14, Bowtie2, RNAmmer v.1.2, tRNAscan-SE v.2.0.12, factoextra v.1.0.7, eggNOG-mapper webserver v.2.1.1.2, clusterProfiler v.4.8.3, ggpubr v.0.6.0, R v.4.3.1.<br><br>For quantification of ribosomal footprints and RNA-Seq reads, a custom script was used (https://github.com/ingolia-lab/RiboSeq)<br><br>Scripts for analysis and figures of high-resolution have been uploaded to zenodo under doi: 10.5281/zenodo.16555392

For manuscripts utilizing custom algorithms or software that are central to the research but not yet described in published literature, software must be made available to editors and reviewers. We strongly encourage code deposition in a community repository (e.g. GitHub). See the Nature Portfolio guidelines for submitting code & software for further information.

Data

Policy information about availability of data

All manuscripts must include a data availability statement. This statement should provide the following information, where applicable:

- Accession codes, unique identifiers, or web links for publicly available datasets
- A description of any restrictions on data availability
- For clinical datasets or third party data, please ensure that the statement adheres to our policy

The RNA-Seq, Ribo-Seq, and mim-tRNA-Seq results obtained in this study have been deposited in the National Centre for Biotechnology Information (NCBI) database under following accession numbers.

RNA-Seq: GSE276076 (https://www.ncbi.nlm.nih.gov/geo/query/acc.cgi?acc=GSE276076)
Ribo-Seq: GSE276078 (https://www.ncbi.nlm.nih.gov/geo/query/acc.cgi?acc=GSE276078)
tRNA-Seq: GSE276080 (https://www.ncbi.nlm.nih.gov/geo/query/acc.cgi?acc=GSE276080)

Research involving human participants, their data, or biological material

Policy information about studies with human participants or human data. See also policy information about sex, gender (identity/presentation), and sexual orientation and race, ethnicity and racism.

Reporting on sex and gender	Not applicable
Reporting on race, ethnicity, or other socially relevant groupings	Not applicable
Population characteristics	Not applicable
Recruitment	Not applicable
Ethics oversight	Note applicable

Note that full information on the approval of the study protocol must also be provided in the manuscript.

Field-specific reporting

Please select the one below that is the best fit for your research. If you are not sure, read the appropriate sections before making your selection.

☒ Life sciences ☐ Behavioural & social sciences ☐ Ecological, evolutionary & environmental sciences

For a reference copy of the document with all sections, see nature.com/documents/nr-reporting-summary-flat.pdf

Life sciences study design

All studies must disclose on these points even when the disclosure is negative.

Sample size	The study focuses on investigating the translation dynamics during the infection course of acanthamoeba castellanii mimivirus. We conducted a time-course virus infection experiments and collected samples at 0 (for mock infection), 2, 4, and 8 hours post infection. For each time point, two independent biological replicates were prepared. When conducting statistical test at gene level, all genes with enough reads were used. For codon-wise comparison, all codons except stop codon were used. Detailed information about the sample numbers is provided in the manuscript. These samples were used for ribosome profiling, RNA-Seq and tRNA-Seq, in line with the standard protocols in the field. No statistical methods were employed to predetermine the sample size. The sample size is similar to those generally employed in the field.
Data exclusions	No data were excluded from the analysis.
Replication	The reproducibility of our experiments was confirmed with two replicates tested for deep sequencing experiments. All experiments were repeatable. Results can be reproduced with the parameters and program provided in the methods section.
Randomization	Randomization is not relevant to this study.
Blinding	Blinding is not applicable to this study.

Reporting for specific materials, systems and methods

We require information from authors about some types of materials, experimental systems and methods used in many studies. Here, indicate whether each material, system or method listed is relevant to your study. If you are not sure if a list item applies to your research, read the appropriate section before selecting a response.

Materials & experimental systems

n/a	Involved in the study
☒	☐ Antibodies
☐	☒ Eukaryotic cell lines
☒	☐ Palaeontology and archaeology
☒	☐ Animals and other organisms
☒	☐ Clinical data
☒	☐ Dual use research of concern
☒	☐ Plants

Methods

n/a	Involved in the study
☒	☐ ChIP-seq
☒	☐ Flow cytometry
☒	☐ MRI-based neuroimaging

Eukaryotic cell lines

Policy information about cell lines and Sex and Gender in Research

Cell line source(s)	Acanthamoeba castellanii, strain Neff [American Type Culture Collection (ATCC), 30010]
Authentication	n.a
Mycoplasma contamination	n.a
Commonly misidentified lines (See ICLAC register)	n.a

Plants

Seed stocks	n.a
Novel plant genotypes	n.a
Authentication	n.a
