# Supplementary figures and images for: A giant virus forms a specialized subcellular environment within its amoeba host for efficient translation
Source: Nat Microbiol. 2026 Jan 9;11(2):584–96. doi: 10.1038/s41564-025-02234-x (PMC12872441; doi:10.1038/s41564-025-02234-x)

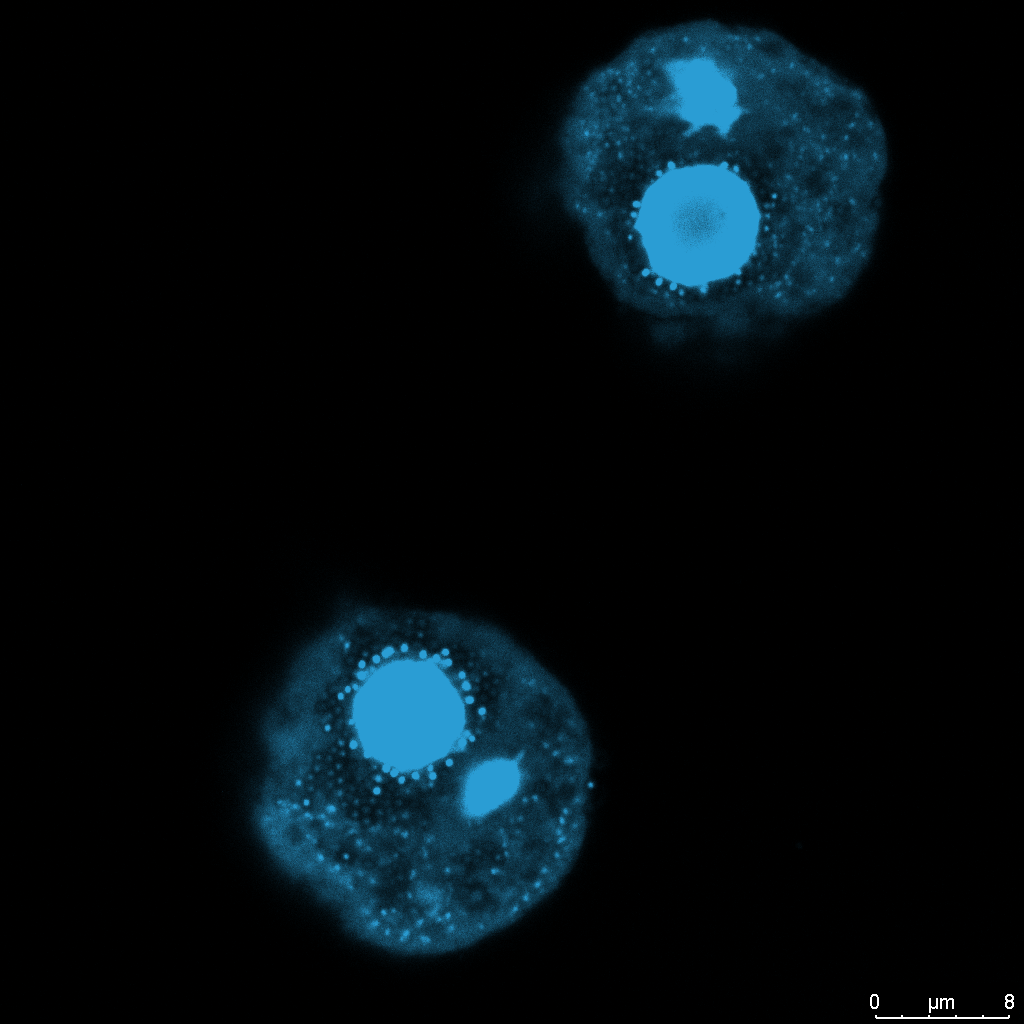

Supplement: Supplementary file 8 — Unprocessed microscopy images for Fig. 5. [file 41564_2025_2234_MOESM8_ESM.zip › Figure5_microscopy/Figure_5B_DAPI.tif]

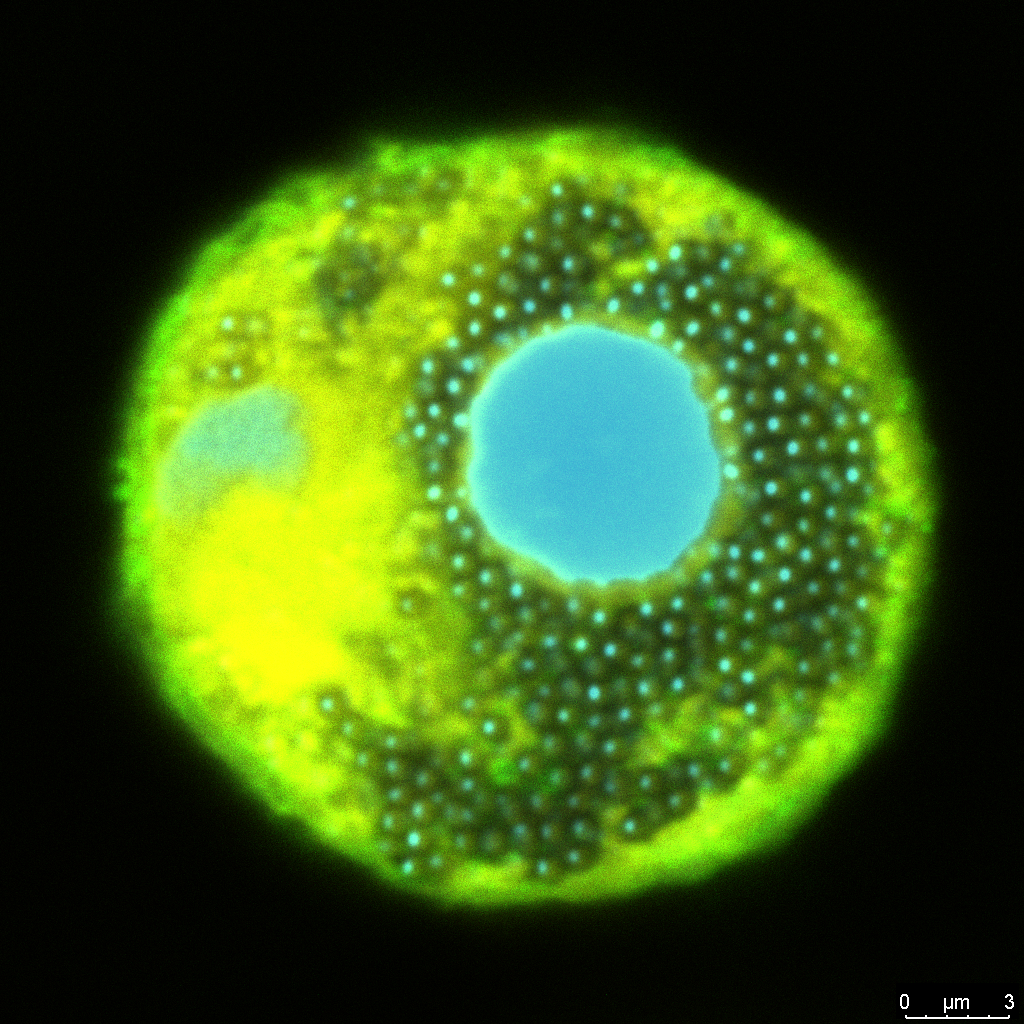

Supplement: Supplementary file 8 — Unprocessed microscopy images for Fig. 5. [file 41564_2025_2234_MOESM8_ESM.zip › Figure5_microscopy/Figure_5A_rRNA_BONCAT_DAPI_overlay.tif]

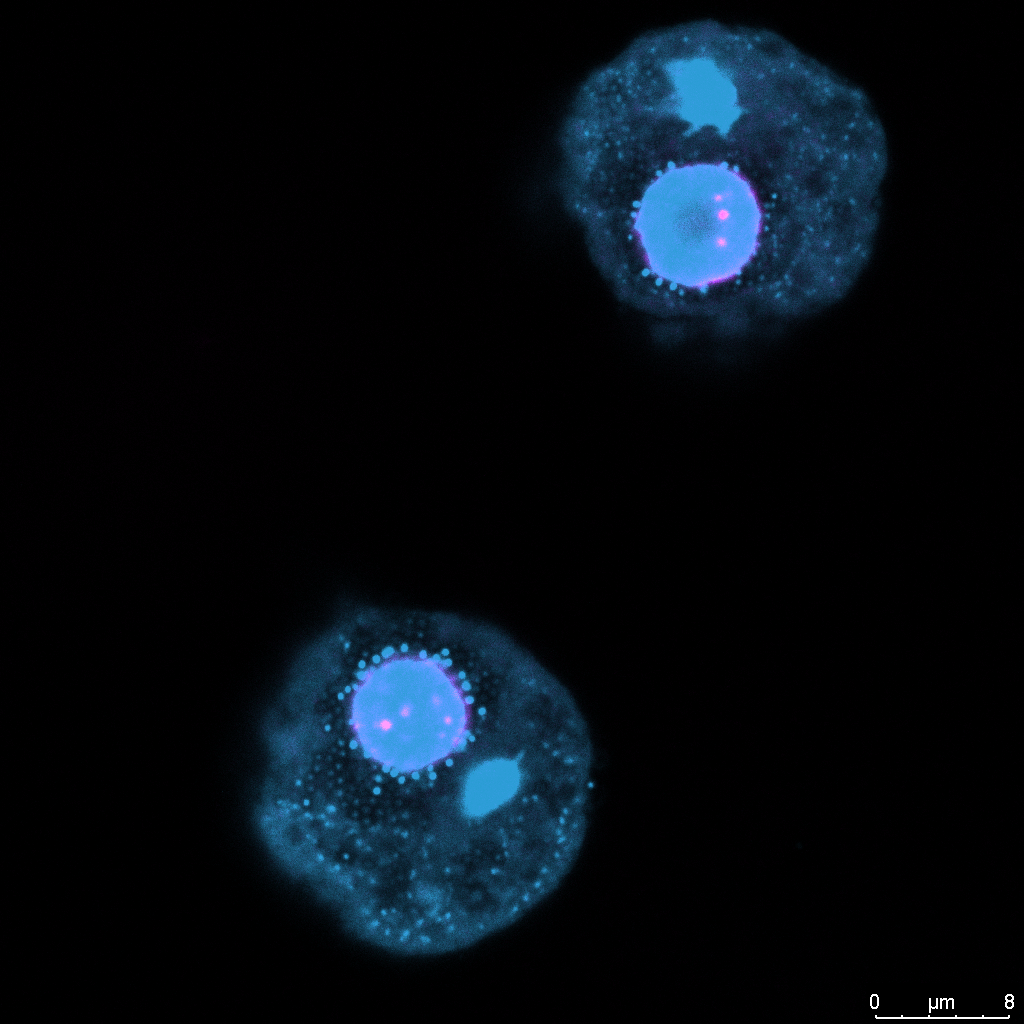

Supplement: Supplementary file 8 — Unprocessed microscopy images for Fig. 5. [file 41564_2025_2234_MOESM8_ESM.zip › Figure5_microscopy/Figure_5B_mRNA_DAPI_overlay.tif]

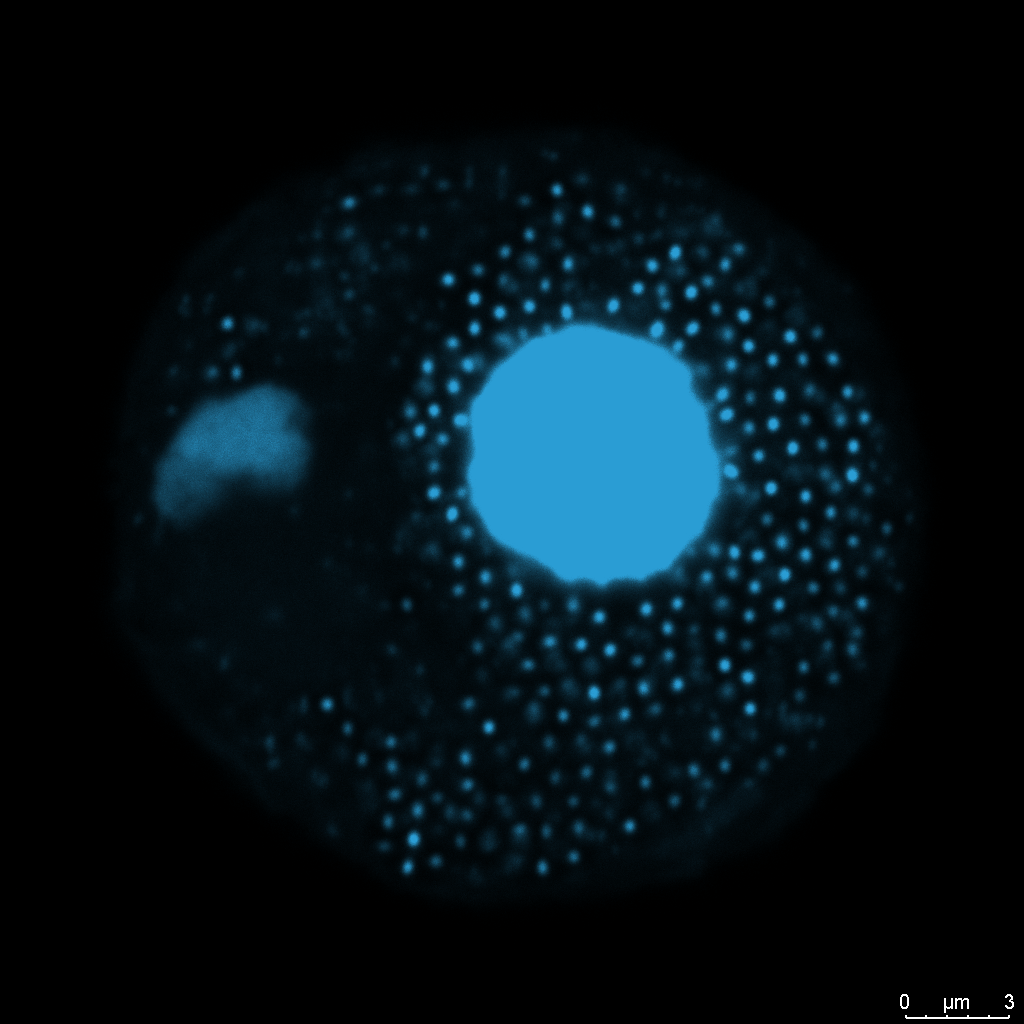

Supplement: Supplementary file 8 — Unprocessed microscopy images for Fig. 5. [file 41564_2025_2234_MOESM8_ESM.zip › Figure5_microscopy/Figure_5A_DAPI.tif]

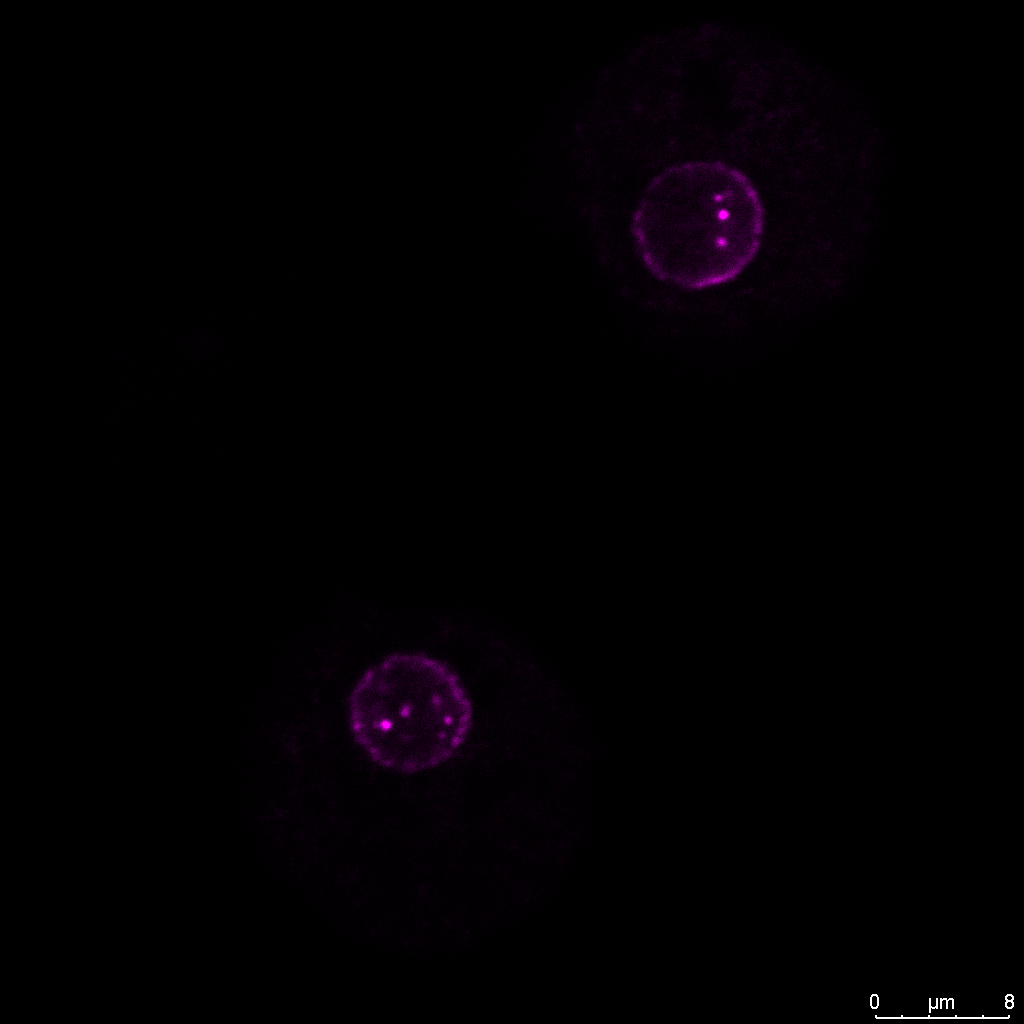

Supplement: Supplementary file 8 — Unprocessed microscopy images for Fig. 5. [file 41564_2025_2234_MOESM8_ESM.zip › Figure5_microscopy/Figure_5B_mRNA.tif]

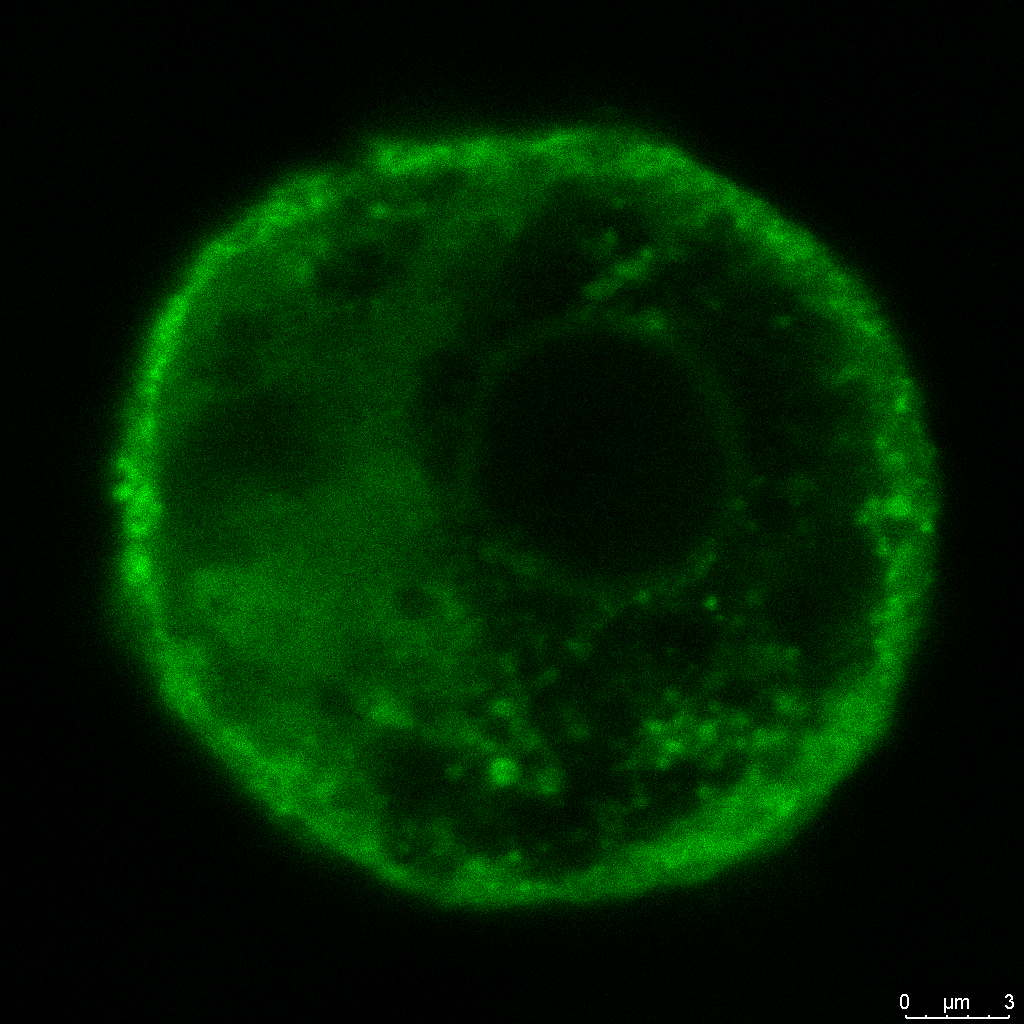

Supplement: Supplementary file 8 — Unprocessed microscopy images for Fig. 5. [file 41564_2025_2234_MOESM8_ESM.zip › Figure5_microscopy/Figure_5A_rRNA.tif]

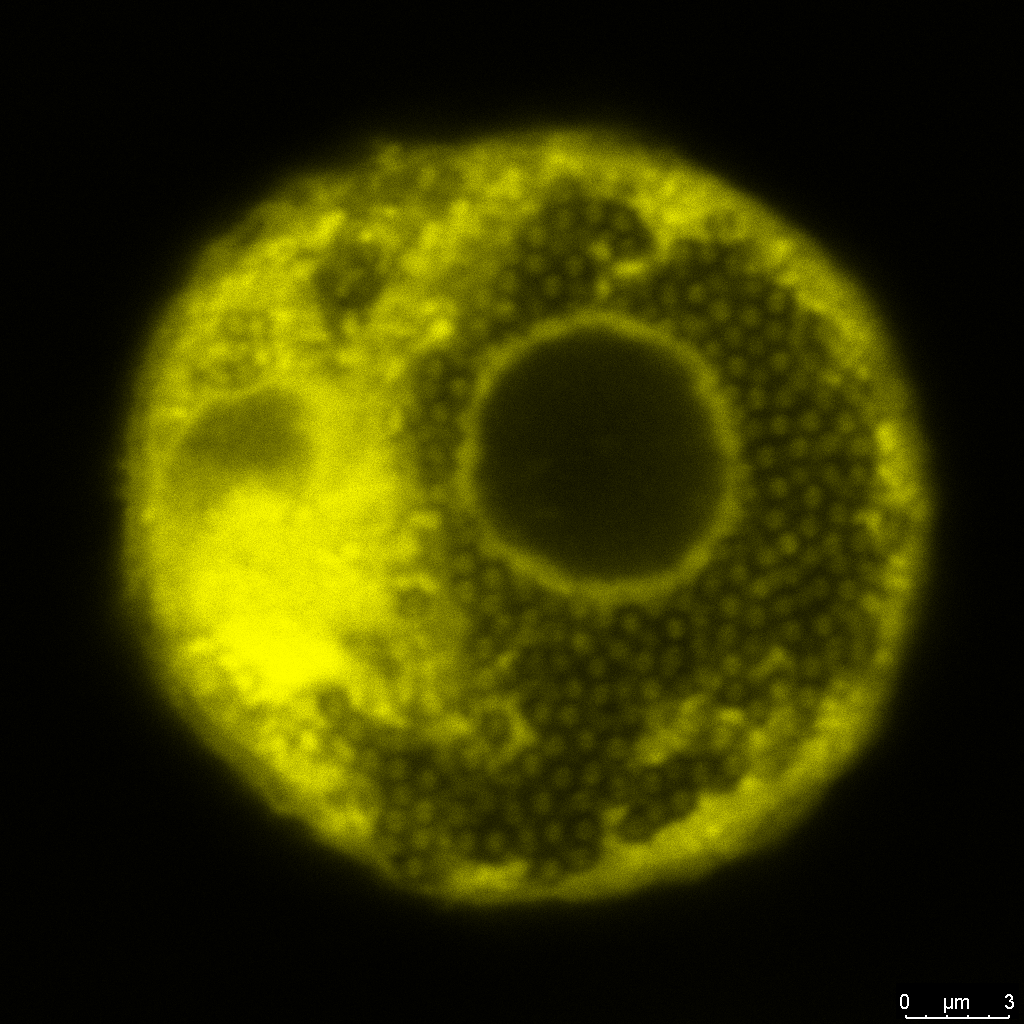

Supplement: Supplementary file 8 — Unprocessed microscopy images for Fig. 5. [file 41564_2025_2234_MOESM8_ESM.zip › Figure5_microscopy/Figure_5A_BONCAT.tif]

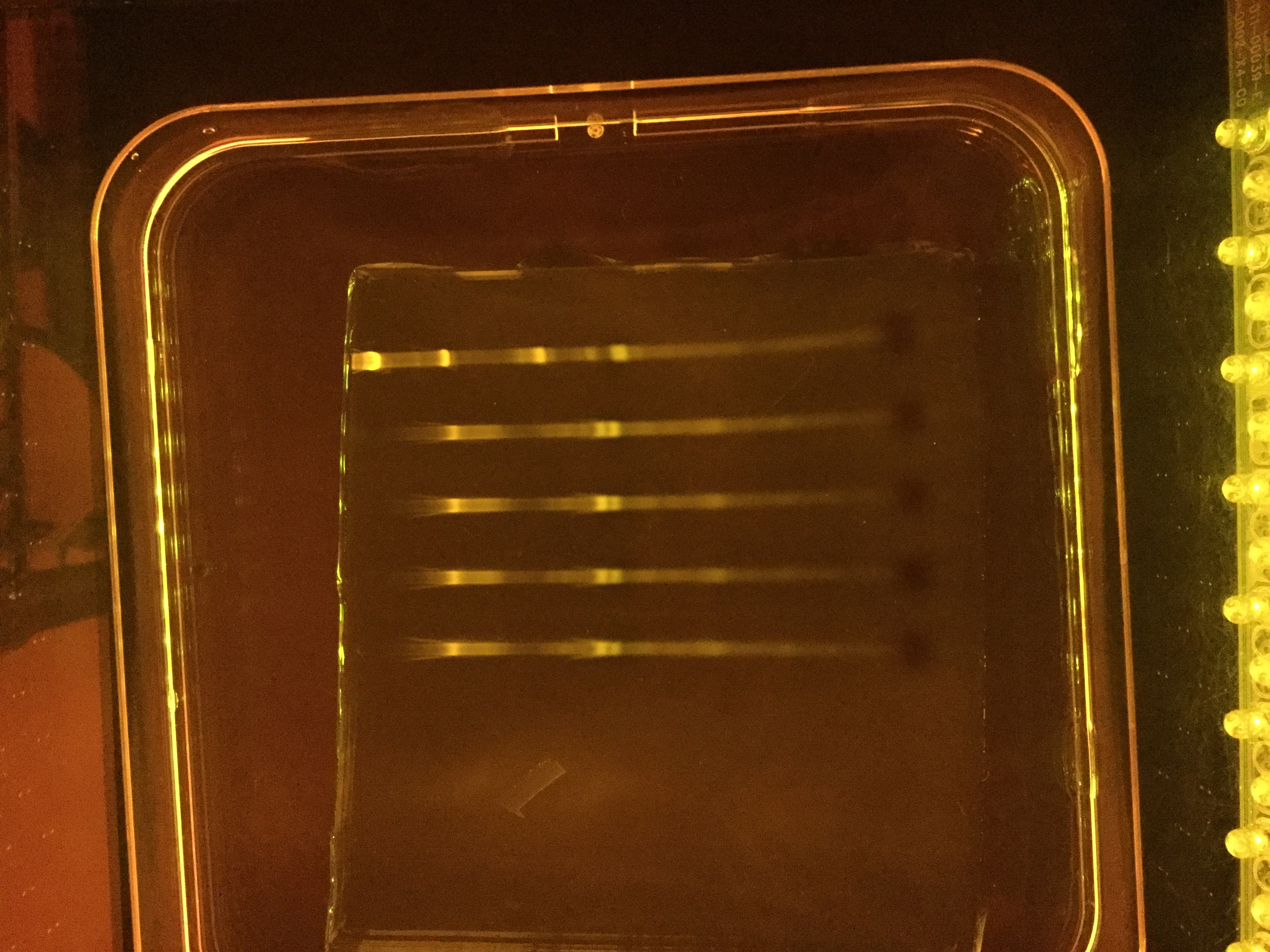

Supplement: Supplementary file 13 — Unprocessed gels for Extended Data Fig. 5a,b. [file 41564_2025_2234_MOESM13_ESM.zip › FigureS5_gel_image/FigureS5A_122322_RT.jpg]

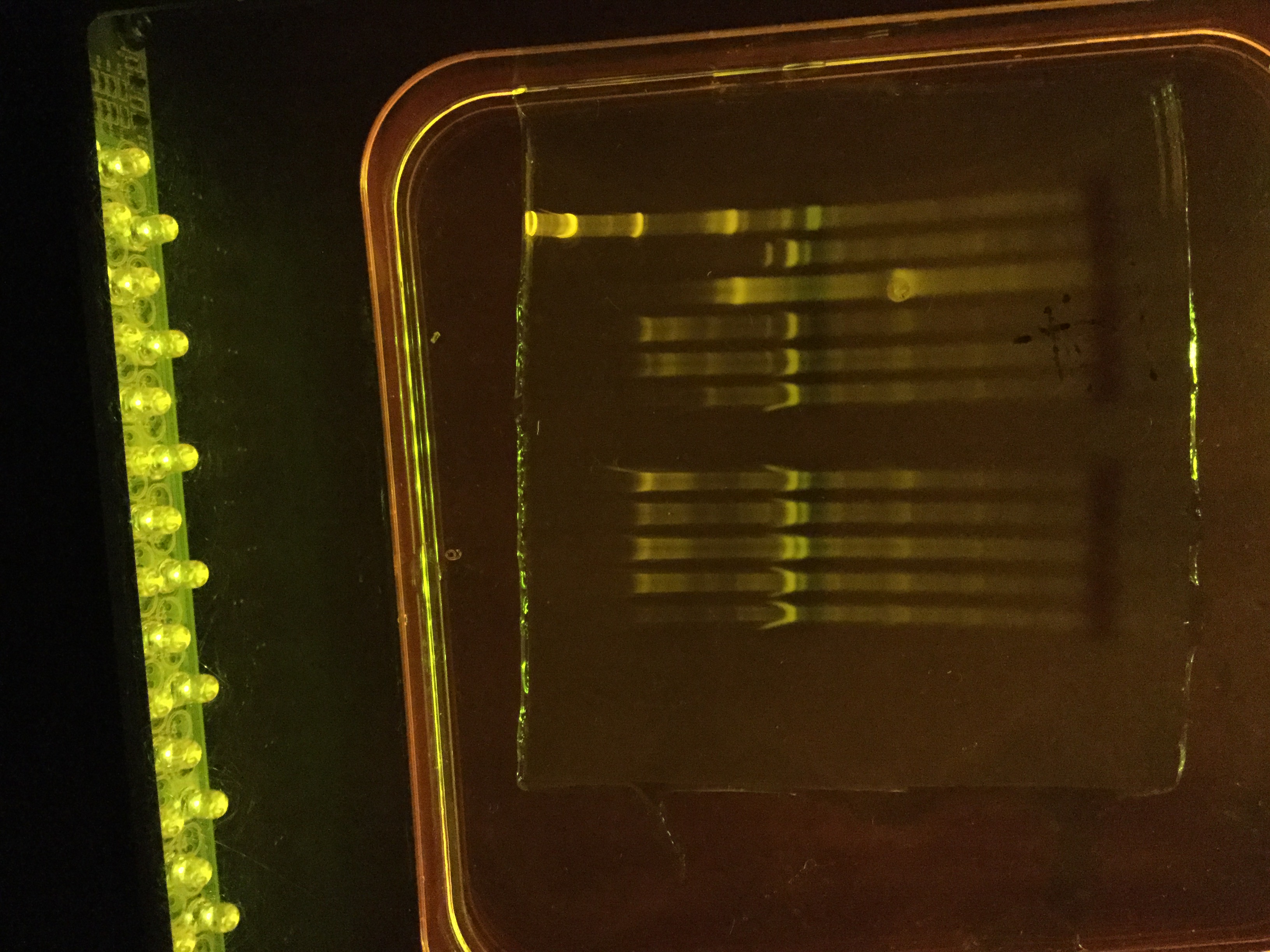

Supplement: Supplementary file 13 — Unprocessed gels for Extended Data Fig. 5a,b. [file 41564_2025_2234_MOESM13_ESM.zip › FigureS5_gel_image/FigureS5B_121622_RT_condition_check.jpg]
